# Crystal structure of 1-benzyl-2-hy­droxy-5-oxopyrrolidin-3-yl acetate

**DOI:** 10.1107/S2056989015013353

**Published:** 2015-07-17

**Authors:** Ignez Caracelli, Julio Zukerman-Schpector, Hélio A. Stefani, Bakhat Ali, Edward R. T. Tiekink

**Affiliations:** a Departmento de Física, Universidade Federal de São Carlos, 13565-905 São Carlos, SP, Brazil; b Departmento de Química, Universidade Federal de São Carlos, 13565-905 São Carlos, SP, Brazil; c Departamento de Farmácia, Faculdade de Ciências Farmacêuticas, Universidade de São Paulo, 05508-900 São Paulo-SP, Brazil; d Department of Chemistry, University of Malaya, 50603 Kuala Lumpur, Malaysia

**Keywords:** crystal structure, oxopyrrolidin-3-yl, hydrogen bonding, conformation

## Abstract

In the title compound, C_13_H_15_NO_4_, the oxopyrrolidin-3-yl ring has an envelope conformation, with the C atom bearing the acetate group being the flap. The acetate and phenyl groups are inclined with respect to the central ring, forming dihedral angles of 50.20 (12) and 87.40 (9)°, respectively, with the least-squares plane through the ring. The dihedral angle between the acetate group and the phenyl ring is 63.22 (8)°, indicating a twisted conformation in the mol­ecule. In the crystal, supra­molecular chains along the *b* axis are formed by (hy­droxy)O—H⋯O(ring carbon­yl) hydrogen bonds. The chains are consolidated into the three-dimensional architecture by C—H⋯O inter­actions.

## Related literature   

For the synthesis of symmetrical 1,4-dioxanes, including the title compound, *via* Lewis-acid-catalysed *N*-acyl­iminium ion cyclo­dimerization, and for a related structure, see: Ali *et al.* (2015 ▸).
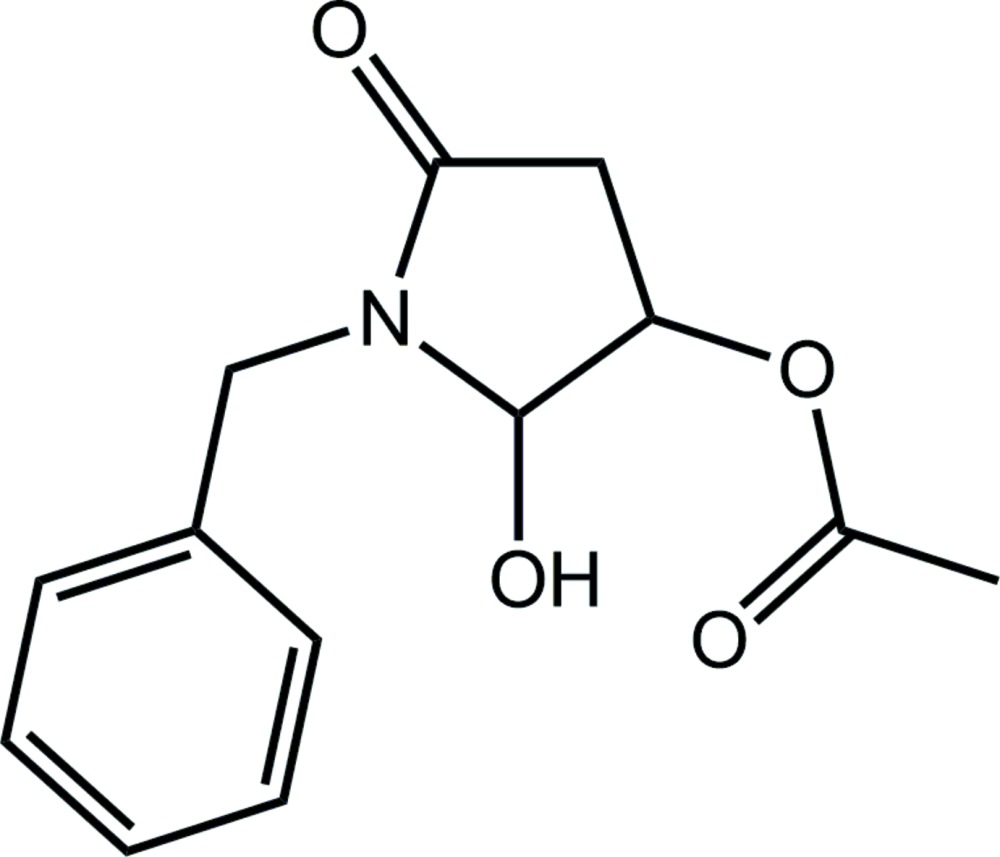


## Experimental   

### Crystal data  

C_13_H_15_NO_4_
*M* *_r_* = 249.26
Orthorhombic, 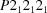

*a* = 26.504 (2) Å
*b* = 6.3668 (5) Å
*c* = 7.6040 (6) Å
*V* = 1283.14 (17) Å^3^
*Z* = 4
Mo *K*α radiation
μ = 0.10 mm^−1^
*T* = 293 K
0.56 × 0.40 × 0.36 mm

### Data collection  

Bruker APEXII CCD diffractometer
Absorption correction: multi-scan (*SADABS*; Sheldrick, 1996 ▸) *T*_min_ = 0.673, *T*_max_ = 0.745
4917 measured reflections
2246 independent reflections
2087 reflections with *I* > 2σ(*I*)
*R*_int_ = 0.014

### Refinement  

*R*[*F* ^2^ > 2σ(*F* ^2^)] = 0.034
*wR*(*F* ^2^) = 0.093
*S* = 1.06
2246 reflections
165 parameters
H-atom parameters constrained
Δρ_max_ = 0.10 e Å^−3^
Δρ_min_ = −0.18 e Å^−3^

### 

Data collection: *APEX2* (Bruker, 2009 ▸); cell refinement: *SAINT* (Bruker, 2009 ▸); data reduction: *SAINT*; program(s) used to solve structure: *SIR2014* (Burla *et al.*, 2015 ▸); program(s) used to refine structure: *SHELXL2014* (Sheldrick, 2015 ▸); molecular graphics: *ORTEP-3 for Windows* (Farrugia, 2012 ▸) and *DIAMOND* (Brandenburg, 2006 ▸); software used to prepare material for publication: *MarvinSketch* (ChemAxon, 2010 ▸) and *publCIF* (Westrip, 2010 ▸).

## Supplementary Material

Crystal structure: contains datablock(s) I, New_Global_Publ_Block. DOI: 10.1107/S2056989015013353/hg5452sup1.cif

Structure factors: contains datablock(s) I. DOI: 10.1107/S2056989015013353/hg5452Isup2.hkl

Supporting information file. DOI: 10.1107/S2056989015013353/hg5452Isup3.cml

**Fig. 1.**
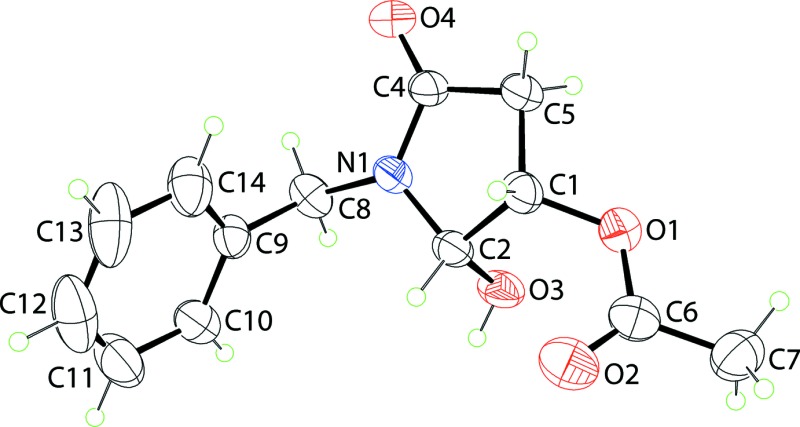
The molecular structure of the title compound showing the atom-labelling scheme and displacement ellipsoids at the 35% probability level.

**Fig. 2.**
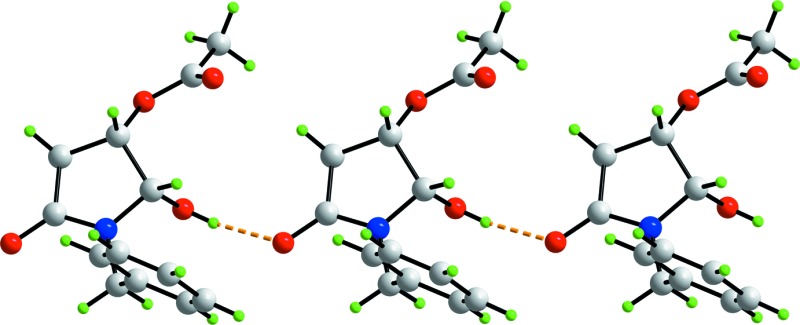
A view of the supramolecular chain along the b axis mediated by O—H···O hydrogen bonding shown as orange dashed lines.

**Fig. 3.**
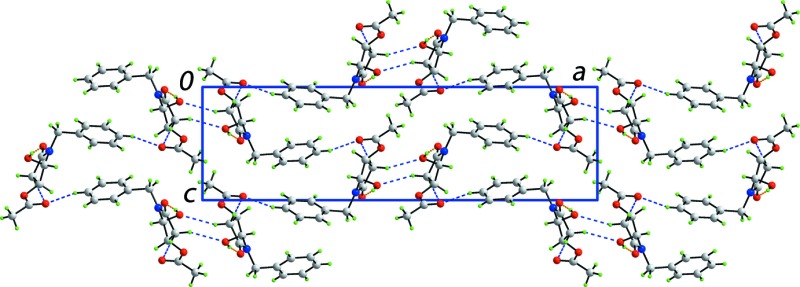
A view in projection down the b axis of the unit-cell contents. The O—H···O and C—H···O interactions shown as orange and blue dashed lines, respectively.

. DOI: 10.1107/S2056989015013353/hg5452fig1.tif
The mol­ecular structure of the title compound showing the atom-labelling scheme and displacement ellipsoids at the 35% probability level.

b . DOI: 10.1107/S2056989015013353/hg5452fig2.tif
A view of the supra­molecular chain along the *b* axis mediated by O—H⋯O hydrogen bonding shown as orange dashed lines.

b . DOI: 10.1107/S2056989015013353/hg5452fig3.tif
A view in projection down the *b* axis of the unit-cell contents. The O—H⋯O and C—H⋯O inter­actions shown as orange and blue dashed lines, respectively.

CCDC reference: 1412190

Additional supporting information:  crystallographic information; 3D view; checkCIF report

## Figures and Tables

**Table 1:** Hydrogen-bond geometry (, )

*D*H*A*	*D*H	H*A*	*D* *A*	*D*H*A*
O3H3*O*O4^i^	0.82	1.86	2.671(2)	170
C5H5*A*O2^ii^	0.97	2.49	3.452(3)	170
C5H5*B*O3^iii^	0.97	2.51	3.437(3)	160
C12H12O2^iv^	0.93	2.54	3.421(4)	158

Symmetry code: (i) 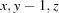; (ii) 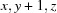; (iii) 
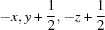
; (iv) 
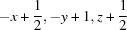
.

## supplementary crystallographic information

### S3. Experimental

The title compound was prepared as described in the literature (Ali *et al.*, 2015) and crystals were obtained from the slow evaporation of its methanol solution.

### S4. Refinement

Carbon-bound H-atoms were placed in calculated positions (C—H = 0.93–0.98 Å) and were included in the refinement in the riding model approximation, with *U*_iso_(H) = 1.2–1.5*U*_eq_(C). The O-bound H-atom was treated similarly with O—H = 0.82 Å and *U*_iso_(H) = 1.5*U*_eq_(O).

### Crystal data

**Table:**

C_13_H_15_NO_4_	*D*_x_ = 1.290 Mg m^−^^3^
*M**_r_* = 249.26	Mo *K*α radiation, λ = 0.71073 Å
Orthorhombic, *P*2_1_2_1_2_1_	Cell parameters from 3310 reflections
*a* = 26.504 (2) Å	θ = 2.8–25.3°
*b* = 6.3668 (5) Å	µ = 0.10 mm^−^^1^
*c* = 7.6040 (6) Å	*T* = 293 K
*V* = 1283.14 (17) Å^3^	Block, colourless
*Z* = 4	0.56 × 0.40 × 0.36 mm
*F*(000) = 528	

### Data collection

**Table:**

Bruker APEXII CCD diffractometer	2087 reflections with *I* > 2σ(*I*)
φ and ω scans	*R*_int_ = 0.014
Absorption correction: multi-scan (*SADABS*; Sheldrick, 1996)	θ_max_ = 25.3°, θ_min_ = 2.8°
*T*_min_ = 0.673, *T*_max_ = 0.745	*h* = −31→26
4917 measured reflections	*k* = −5→7
2246 independent reflections	*l* = −9→7

### Refinement

**Table:**

Refinement on *F*^2^	0 restraints
Least-squares matrix: full	Hydrogen site location: inferred from neighbouring sites
*R*[*F*^2^ > 2σ(*F*^2^)] = 0.034	H-atom parameters constrained
*wR*(*F*^2^) = 0.093	*w* = 1/[σ^2^(*F*_o_^2^) + (0.0507*P*)^2^ + 0.1542*P*] where *P* = (*F*_o_^2^ + 2*F*_c_^2^)/3
*S* = 1.06	(Δ/σ)_max_ < 0.001
2246 reflections	Δρ_max_ = 0.10 e Å^−^^3^
165 parameters	Δρ_min_ = −0.18 e Å^−^^3^

### Special details

**Table:**

Geometry. All e.s.d.'s (except the e.s.d. in the dihedral angle between two l.s. planes) are estimated using the full covariance matrix. The cell e.s.d.'s are taken into account individually in the estimation of e.s.d.'s in distances, angles and torsion angles; correlations between e.s.d.'s in cell parameters are only used when they are defined by crystal symmetry. An approximate (isotropic) treatment of cell e.s.d.'s is used for estimating e.s.d.'s involving l.s. planes.

### Fractional atomic coordinates and isotropic or equivalent isotropic displacement parameters (Å^2^)

**Table:**

	*x*	*y*	*z*	*U*_iso_*/*U*_eq_	
O1	0.04798 (6)	0.8021 (3)	0.0393 (2)	0.0590 (4)	
O2	0.10250 (7)	0.5494 (3)	−0.0319 (3)	0.0797 (6)	
O3	0.05817 (6)	0.6595 (2)	0.3698 (2)	0.0626 (5)	
H3O	0.0678	0.5486	0.4131	0.094*	
O4	0.09632 (7)	1.2919 (2)	0.4745 (3)	0.0736 (5)	
N1	0.11194 (6)	0.9434 (3)	0.4315 (3)	0.0528 (5)	
C1	0.08701 (8)	0.8984 (3)	0.1433 (3)	0.0540 (5)	
H1	0.1175	0.9160	0.0717	0.065*	
C2	0.09990 (8)	0.7747 (3)	0.3111 (3)	0.0497 (5)	
H2	0.1291	0.6831	0.2924	0.060*	
C4	0.09357 (8)	1.1311 (3)	0.3844 (3)	0.0547 (5)	
C5	0.06905 (10)	1.1103 (4)	0.2074 (4)	0.0614 (6)	
H5A	0.0799	1.2216	0.1289	0.074*	
H5B	0.0326	1.1139	0.2173	0.074*	
C6	0.06079 (10)	0.6229 (4)	−0.0410 (3)	0.0594 (6)	
C7	0.01803 (12)	0.5271 (5)	−0.1385 (4)	0.0792 (8)	
H7A	0.0041	0.4141	−0.0706	0.119*	
H7B	0.0299	0.4738	−0.2492	0.119*	
H7C	−0.0075	0.6314	−0.1589	0.119*	
C8	0.13462 (9)	0.9012 (4)	0.6024 (3)	0.0605 (6)	
H8A	0.1153	0.7925	0.6613	0.073*	
H8B	0.1328	1.0272	0.6738	0.073*	
C9	0.18888 (8)	0.8323 (4)	0.5891 (3)	0.0525 (5)	
C10	0.20395 (10)	0.6420 (4)	0.6570 (4)	0.0707 (7)	
H10	0.1803	0.5528	0.7079	0.085*	
C11	0.25400 (13)	0.5831 (6)	0.6500 (5)	0.0973 (11)	
H11	0.2643	0.4563	0.6991	0.117*	
C12	0.28864 (12)	0.7121 (8)	0.5703 (5)	0.1080 (14)	
H12	0.3222	0.6711	0.5627	0.130*	
C13	0.27379 (11)	0.9002 (9)	0.5023 (4)	0.1085 (14)	
H13	0.2974	0.9877	0.4491	0.130*	
C14	0.22412 (10)	0.9614 (6)	0.5120 (4)	0.0799 (9)	
H14	0.2143	1.0905	0.4662	0.096*	

### Atomic displacement parameters (Å^2^)

**Table:**

	*U*^11^	*U*^22^	*U*^33^	*U*^12^	*U*^13^	*U*^23^
O1	0.0530 (9)	0.0571 (9)	0.0668 (10)	0.0025 (7)	−0.0072 (7)	0.0012 (8)
O2	0.0751 (12)	0.0781 (12)	0.0861 (13)	0.0164 (10)	0.0149 (10)	0.0015 (11)
O3	0.0543 (8)	0.0431 (8)	0.0902 (12)	−0.0007 (7)	0.0038 (8)	0.0173 (8)
O4	0.0824 (12)	0.0420 (8)	0.0963 (13)	−0.0005 (8)	−0.0194 (10)	0.0025 (9)
N1	0.0482 (9)	0.0437 (9)	0.0664 (11)	0.0037 (8)	−0.0082 (9)	0.0093 (8)
C1	0.0446 (11)	0.0507 (12)	0.0667 (13)	−0.0019 (9)	−0.0034 (9)	0.0113 (11)
C2	0.0414 (10)	0.0410 (10)	0.0667 (13)	0.0048 (9)	0.0002 (9)	0.0066 (10)
C4	0.0470 (11)	0.0385 (10)	0.0785 (15)	−0.0031 (9)	−0.0072 (10)	0.0097 (11)
C5	0.0593 (13)	0.0433 (11)	0.0817 (16)	0.0010 (10)	−0.0149 (12)	0.0136 (11)
C6	0.0685 (15)	0.0562 (12)	0.0535 (12)	0.0004 (12)	0.0092 (11)	0.0130 (11)
C7	0.103 (2)	0.0685 (16)	0.0663 (15)	−0.0102 (16)	−0.0066 (16)	−0.0010 (13)
C8	0.0590 (13)	0.0645 (14)	0.0578 (12)	0.0100 (11)	−0.0006 (10)	0.0109 (11)
C9	0.0502 (11)	0.0596 (13)	0.0478 (10)	−0.0003 (10)	−0.0082 (9)	0.0043 (11)
C10	0.0658 (15)	0.0616 (14)	0.0847 (17)	0.0089 (12)	−0.0054 (13)	0.0060 (15)
C11	0.080 (2)	0.099 (2)	0.113 (3)	0.038 (2)	−0.021 (2)	−0.009 (2)
C12	0.0561 (16)	0.185 (4)	0.083 (2)	0.029 (2)	−0.0068 (16)	−0.022 (3)
C13	0.0540 (16)	0.197 (4)	0.0745 (19)	−0.027 (2)	−0.0063 (14)	0.025 (3)
C14	0.0660 (16)	0.103 (2)	0.0712 (16)	−0.0153 (15)	−0.0117 (13)	0.0294 (17)

### Geometric parameters (Å, º)

**Table:**

O1—C6	1.338 (3)	C7—H7A	0.9600
O1—C1	1.439 (3)	C7—H7B	0.9600
O2—C6	1.202 (3)	C7—H7C	0.9600
O3—C2	1.400 (3)	C8—C9	1.507 (3)
O3—H3O	0.8200	C8—H8A	0.9700
O4—C4	1.234 (3)	C8—H8B	0.9700
N1—C4	1.339 (3)	C9—C14	1.375 (4)
N1—C2	1.447 (3)	C9—C10	1.376 (4)
N1—C8	1.457 (3)	C10—C11	1.380 (4)
C1—C5	1.512 (3)	C10—H10	0.9300
C1—C2	1.538 (3)	C11—C12	1.373 (5)
C1—H1	0.9800	C11—H11	0.9300
C2—H2	0.9800	C12—C13	1.363 (6)
C4—C5	1.501 (4)	C12—H12	0.9300
C5—H5A	0.9700	C13—C14	1.375 (5)
C5—H5B	0.9700	C13—H13	0.9300
C6—C7	1.486 (4)	C14—H14	0.9300
			
C6—O1—C1	115.57 (18)	C6—C7—H7B	109.5
C2—O3—H3O	109.5	H7A—C7—H7B	109.5
C4—N1—C2	114.41 (18)	C6—C7—H7C	109.5
C4—N1—C8	123.6 (2)	H7A—C7—H7C	109.5
C2—N1—C8	121.22 (18)	H7B—C7—H7C	109.5
O1—C1—C5	109.34 (18)	N1—C8—C9	112.81 (19)
O1—C1—C2	113.42 (17)	N1—C8—H8A	109.0
C5—C1—C2	105.0 (2)	C9—C8—H8A	109.0
O1—C1—H1	109.6	N1—C8—H8B	109.0
C5—C1—H1	109.6	C9—C8—H8B	109.0
C2—C1—H1	109.6	H8A—C8—H8B	107.8
O3—C2—N1	111.19 (18)	C14—C9—C10	119.3 (2)
O3—C2—C1	110.93 (17)	C14—C9—C8	120.2 (2)
N1—C2—C1	101.18 (17)	C10—C9—C8	120.5 (2)
O3—C2—H2	111.1	C9—C10—C11	120.3 (3)
N1—C2—H2	111.1	C9—C10—H10	119.9
C1—C2—H2	111.1	C11—C10—H10	119.9
O4—C4—N1	124.8 (2)	C12—C11—C10	119.8 (3)
O4—C4—C5	126.6 (2)	C12—C11—H11	120.1
N1—C4—C5	108.6 (2)	C10—C11—H11	120.1
C4—C5—C1	103.40 (18)	C13—C12—C11	120.0 (3)
C4—C5—H5A	111.1	C13—C12—H12	120.0
C1—C5—H5A	111.1	C11—C12—H12	120.0
C4—C5—H5B	111.1	C12—C13—C14	120.3 (3)
C1—C5—H5B	111.1	C12—C13—H13	119.8
H5A—C5—H5B	109.0	C14—C13—H13	119.8
O2—C6—O1	122.6 (2)	C13—C14—C9	120.3 (3)
O2—C6—C7	124.8 (3)	C13—C14—H14	119.9
O1—C6—C7	112.6 (2)	C9—C14—H14	119.9
C6—C7—H7A	109.5		
			
C6—O1—C1—C5	173.36 (18)	O1—C1—C5—C4	146.44 (18)
C6—O1—C1—C2	−69.8 (2)	C2—C1—C5—C4	24.4 (2)
C4—N1—C2—O3	−97.9 (2)	C1—O1—C6—O2	−2.1 (3)
C8—N1—C2—O3	72.3 (2)	C1—O1—C6—C7	177.2 (2)
C4—N1—C2—C1	20.0 (2)	C4—N1—C8—C9	−119.2 (2)
C8—N1—C2—C1	−169.84 (18)	C2—N1—C8—C9	71.6 (3)
O1—C1—C2—O3	−27.8 (3)	N1—C8—C9—C14	59.4 (3)
C5—C1—C2—O3	91.5 (2)	N1—C8—C9—C10	−121.9 (2)
O1—C1—C2—N1	−145.88 (18)	C14—C9—C10—C11	1.0 (4)
C5—C1—C2—N1	−26.6 (2)	C8—C9—C10—C11	−177.7 (3)
C2—N1—C4—O4	174.5 (2)	C9—C10—C11—C12	−1.9 (5)
C8—N1—C4—O4	4.6 (4)	C10—C11—C12—C13	1.6 (6)
C2—N1—C4—C5	−4.9 (3)	C11—C12—C13—C14	−0.4 (6)
C8—N1—C4—C5	−174.8 (2)	C12—C13—C14—C9	−0.6 (5)
O4—C4—C5—C1	167.7 (2)	C10—C9—C14—C13	0.3 (4)
N1—C4—C5—C1	−13.0 (3)	C8—C9—C14—C13	179.0 (3)

### Hydrogen-bond geometry (Å, º)

**Table:**

*D*—H···*A*	*D*—H	H···*A*	*D*···*A*	*D*—H···*A*
O3—H3*O*···O4^i^	0.82	1.86	2.671 (2)	170
C5—H5*A*···O2^ii^	0.97	2.49	3.452 (3)	170
C5—H5*B*···O3^iii^	0.97	2.51	3.437 (3)	160
C12—H12···O2^iv^	0.93	2.54	3.421 (4)	158

Symmetry codes: (i) *x*, *y*−1, *z*; (ii) *x*, *y*+1, *z*; (iii) −*x*, *y*+1/2, −*z*+1/2; (iv) −*x*+1/2, −*y*+1, *z*+1/2.
