# Supplementary figures and images for: Crystal structure of 1-benzyl-2-hy­droxy-5-oxopyrrolidin-3-yl acetate
Source: Acta Crystallogr E Crystallogr Commun. 2015 Jul 17;71(Pt 8):o582–3. doi: 10.1107/S2056989015013353 (PMC4571408; doi:10.1107/S2056989015013353)

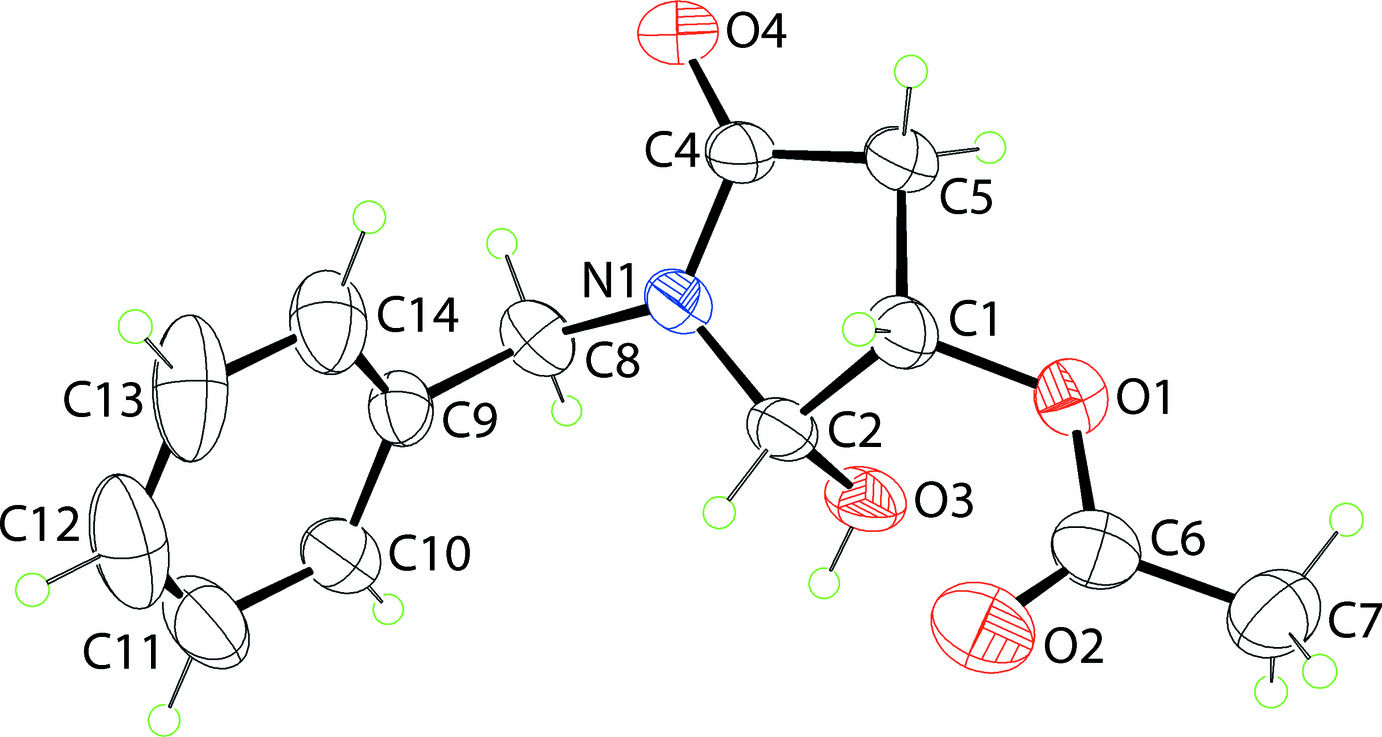

Supplement: Supplementary file 4 [file e-71-0o582-fig1.tif]

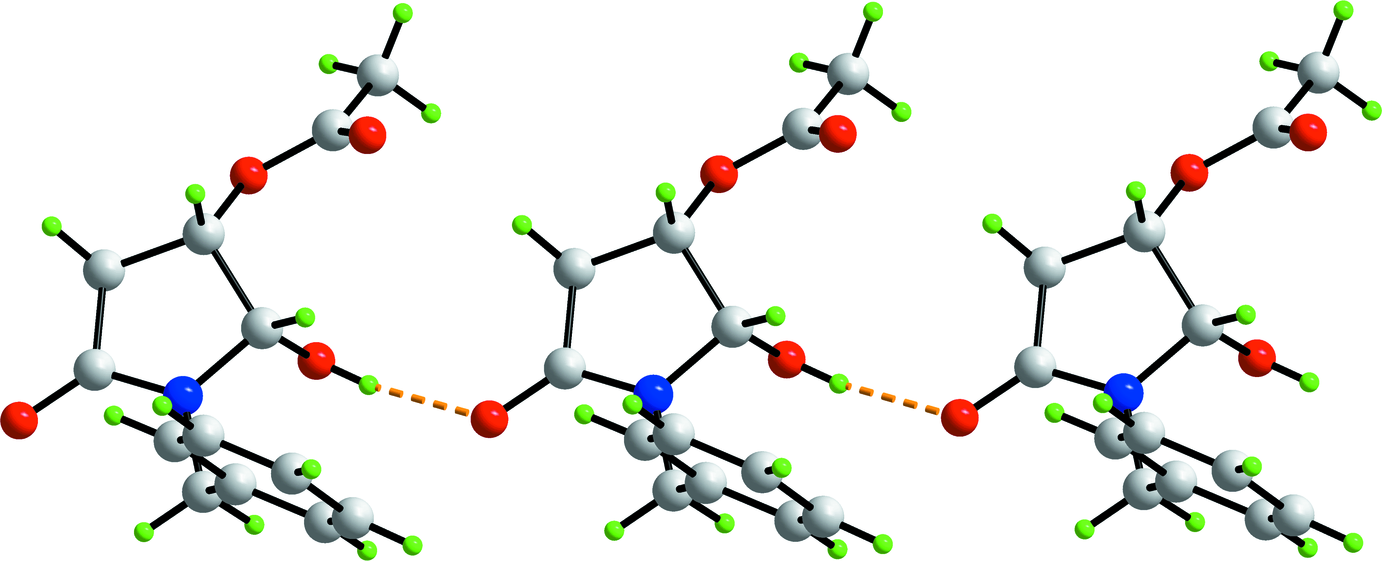

Supplement: Supplementary file 5 [file e-71-0o582-fig2.tif]

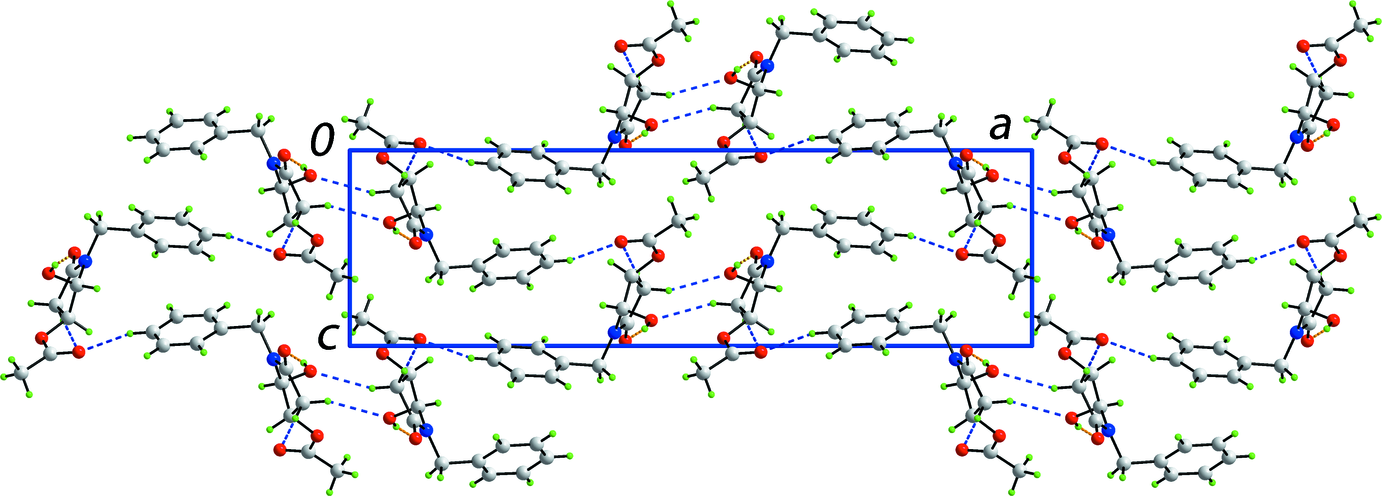

Supplement: Supplementary file 6 [file e-71-0o582-fig3.tif]
